# Transcranial Direct Current Stimulation as a Treatment Tool for Mild Traumatic Brain Injury

**DOI:** 10.3390/brainsci11060806

**Published:** 2021-06-18

**Authors:** Thorsten Rudroff, Craig D. Workman

**Affiliations:** 1 Department of Health and Human Physiology, University of Iowa, Iowa City, IA 52242, USA; craig-workman@uiowa.edu; 2 Department of Neurology, University of Iowa Health Clinics, Iowa City, IA 52242, USA

**Keywords:** tDCS, mild traumatic brain injury, concussion

## Abstract

Mild traumatic brain injury (mTBI) has been defined as a transient (<24 h) condition of confusion and/or loss of consciousness for less than 30 min after brain injury and can result in short- and long-term motor and cognitive impairments. Recent studies have documented the therapeutic potential of non-invasive neuromodulation techniques for the enhancement of cognitive and motor function in mTBI. Alongside repetitive transcranial magnetic stimulation (rTMS), the main technique used for this purpose is transcranial direct current stimulation (tDCS). The focus of this review was to provide a detailed, comprehensive (i.e., both cognitive and motor impairment) overview of the literature regarding therapeutic tDCS paradigms after mTBI. A publication search of the PubMed, Scopus, CINAHL, and PsycINFO databases was performed to identify records that applied tDCS in mTBI. The publication search yielded 14,422 records from all of the databases, however, only three met the inclusion criteria and were included in the final review. Based on the review, there is limited evidence of tDCS improving cognitive and motor performance. Surprisingly, there were only three studies that used tDCS in mTBI, which highlights an urgent need for more research to provide additional insights into ideal therapeutic brain targets and optimized stimulation parameters.

## 1. Introduction

Mild traumatic brain injury (mTBI; sometimes referred to as a concussion) has been defined as a transient (<24 h) condition of confusion and/or loss of consciousness for less than 30 min after brain injury [1] and might be the result of diffuse brain injury that can affect motor and cognitive functions [2,3,4,5]. Cognitive symptoms of mTBI might include confusion, difficulty focusing attention, impaired memory, and reduced visual processing speed [6,7,8]. Recovery from sport-related mTBI is typically assessed using computerized neurocognitive testing, usually within 10 days post-injury [9,10]. A growing body of literature, however, suggests that even in the absence of neurocognitive deficits, motor impairments such as reduced movement speed and difficulties with gait and balance control can persist in the longer term [4,11,12,13].

The sequelae experienced by mTBI patients (e.g., long-term physical, mental, social, or occupational problems) are difficult to observe but can have profound consequences [14,15,16]. Most patients with mTBI experience symptom resolution within 3 months [17]. However, a large proportion experience post-concussion symptoms (PCS) for an extended period [14]. The symptoms of PCS include balance problems, headache, dizziness, fatigue, sleep disturbance, irritability, difficulties with concentration, memory loss, stress intolerance, light and sound sensitivity, anxiety, and depressed mood [18]. Such prolonged post-injury effects are referred to as persistent post-concussion syndrome (PPCS). Along with changes in emotional regulation, cognitive dysfunction (characterized by impaired concentration, attention, memory, and/or executive function) are also prominent features of the clinical profile of PPCS [18]. It has been hypothesized that PPCS is secondary to microstructural brain damage from shearing injury, which is undetectable with conventional imaging techniques and might underpin the functional and cognitive deficits [19,20]. Using diffusion tensor imaging to measure white matter integrity [21], studies have revealed poorer structural integrity of white matter [22] and altered structural fiber integrity in the corpus callosum of patients with mTBI [23,24,25] and moderate TBI [24,25,26], potentially from brain injury-induced demyelination [21]. The brain regions typically affected by concussion most commonly include the mesial and deeper regions, such as the hippocampus and corpus callosum [19]. This injury “preference” would justify memory complaints reported in post-concussion patients. Additionally, the prefrontal cortex represents another frequently involved brain area, which would account for the persistent executive function deficits in PPCS [2,27]. Specifically, this might explain, at least in part, why some patients have trouble following instructions and performing tasks that were routine before their trauma [28].

Additionally, because patients with mTBI generally do not present with overt structural brain lesions on routine magnetic resonance imaging (MRI) or computed tomography scans, neurochemical changes have also been proposed to account for the slight, but persistent, deficits reported in this population. Such changes have been directly and indirectly investigated via magnetic resonance spectroscopy (MRS) and non-invasive brain stimulation (NIBS) techniques, respectively. MRS studies have postulated a strong link between cognitive impairments and metabolite alterations in patients with mTBI [29]. Specifically, alterations in total choline, *N*-acetylaspartate + *N*-cetylaspartylglutamate, creatine + phosphocreatine, and glutamate + glutamine concentrations have all been evaluated and have revealed promising associations with executive function [30]. Furthermore, recent technological advances have permitted in vivo detection of gamma-aminobutyric acid (GABA) [31,32], which is a promising indicator for metabolic disruption after mTBI [31,32]. Even though MRS studies in humans have thus far failed to reveal differences in GABA concentrations between patients with mTBI and healthy controls [32], altered GABA concentrations after TBI have been reported in animal studies [31,33].

Interestingly, NIBS studies have reported changes in GABA receptor activity in the human motor cortex. Several studies have used transcranial magnetic stimulation (TMS) protocols to investigate the neurochemical mechanisms underpinning mTBI. In particular, TMS measures such as long-interval intracortical inhibition (LICI) and corticomotor silent period (CSP), allows an evaluation of GABA_B_ receptor activity [32] and recent TMS studies have revealed alterations of these parameters in athletes with recurrent mTBI. Most of these studies found enhanced LICI [32,34,35,36] and prolonged CSP duration [32,34,36,37,38], indicating increased activity in the GABA_B_ receptor system and intracortical inhibition-excitation imbalance [39]. In these patients, higher GABA_B_ receptor activity has been coupled with decreased long-term potentiation-like plasticity and motor learning [35].

Recent studies have documented the therapeutic potential of non-invasive neuromodulation for cognitive enhancement [28,40,41,42,43,44,45,46]. Alongside repetitive transcranial magnetic stimulation (rTMS), the main technique used for this purpose is transcranial direct current stimulation (tDCS). tDCS is a NIBS tool that might effectively combine with conventional cognitive and motor rehabilitation to enhance rehabilitation in patients with mTBI. Stimulation administration involves applying electrical currents through the scalp to alter cortical excitability [47] and facilitate neural plasticity. This neuromodulation tool is an especially appealing therapeutic adjunct because it has a relatively low cost, is easy to administer, has an excellent safety record, and a strong potential for in-home use [48,49,50,51,52]. Of particular interest, a proposed mechanism for anodal tDCS (atDCS) excitability enhancement is a reduction in cortical GABA concentration [53,54,55,56]. For example, a recent study found that atDCS reduced GABA_B_-mediated inhibition, indicated by a shortened CSP duration [57]. In addition, there is evidence that tDCS can modulate metabolite concentrations in a polarity-specific manner [56]; atDCS increases cortical excitability, potentially mediated by a decrease in GABA concentration [54,55,56,57] and cathodal tDCS (ctDCS) inhibits cortical excitability, possibly mediated by a reduction in glutamate concentration [53,55,56]. Thus, this NIBS technique would be a promising treatment for patients with pathologically elevated cerebral GABA concentrations, such as mTBI [58].

Several reviews of NIBS in moderate-severe TBI have previously been undertaken [39,59,60,61,62,63]. Specifically, two groups have reviewed the effects of tDCS on attention, memory, inhibitory control, cognitive flexibility [59,60] and another emphasized the effects on motor impairments [61]. However, there are currently no systematic reviews examining the effects of tDCS on cognitive and/or motor impairment after mTBI in athletes. Therefore, the focus of this review was to provide a detailed, comprehensive (i.e., both cognitive and motor impairment) overview of the literature regarding therapeutic tDCS paradigms after mTBI.

## 2. Search Methodology

A publication search of the PubMed, Scopus, CINAHL, and PsycInfo databases was performed to identify records that applied tDCS in mTBI. The PubMed search terms were “transcranial direct current stimulation”[MeSH Terms] OR “transcranial”[All Fields] AND “direct”[All Fields] AND “current”[All Fields] AND “stimulation”[All Fields] OR “transcranial direct current stimulation”[All Fields] OR “tdcs”[All Fields] AND “mild traumatic brain injury”[All Fields] OR “mild”[All Fields] AND “tbi”[All Fields] OR “mild”[All Fields] AND “head injury”[All Fields]. Similar terms were also searched in the other databases. The inclusion criteria for full review were: (1) English-language studies, (2) applied transcranial direct current stimulation to the brain of animals or humans, (3) included a motor, cognitive, or symptomatic outcome measure (i.e., not neuroimaging or motor evoked potentials as the only outcomes), and (4) access to the full text was readily available. Exclusion criteria were: (1) other forms of transcranial electrical stimulation (e.g., transcranial alternating current stimulation, transcranial random noise stimulation, etc.), deep brain stimulation, and repetitive transcranial magnetic stimulation interventions (rTMS, theta burst). The titles and abstracts of the search results were independently examined by two reviewers according to the inclusion/exclusion criteria. Potentially relevant tDCS and mTBI publications were exported and examined in more detail by two reviewers before inclusion in the final review. The bibliographies of retrieved records were also searched for additional publications. A third researcher was consulted in the event of disagreements by the two initial reviewers at any stage of the review process.

## 3. Results

**Figure 1:**
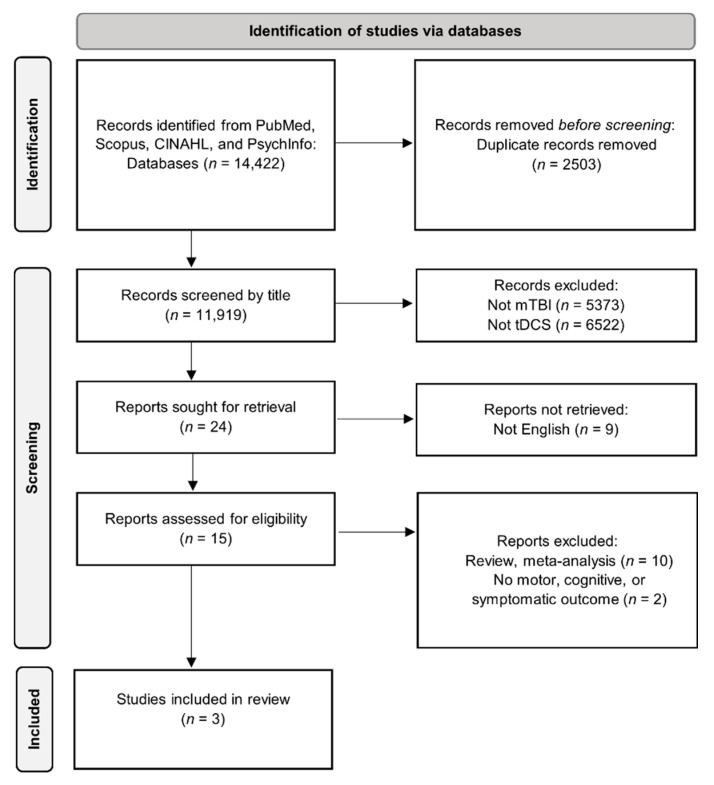
PRISMA flow chart of the literature search, screening, and study inclusion.

Only three publications from the literature search were retained for full review (Figure 1). As mentioned above, headache is a common symptom after mTBI. Pinchuk, et al. [64] retrospectively analyzed the clinical efficacy of tDCS for primary and secondary headache treatment. Among the 90 patients, the study included 44 adolescents aged 11–16 years with chronic post-traumatic headache after mTBI. Three basic localizations (electrode positions (EP)) of stimulating electrodes were used. In the first (1EP) the anode was secured over the frontal pole of the hemisphere less-dominant in motor skill and the cathode was placed on the ipsilateral mastoid process. In 2EP, the anode was located centrally on the forehead at the projection of the interhemispheric fissure, 1.5 cm above the nasal bridge, and the cathode was placed 2 cm superior to the mastoid process of the hemisphere less-dominant in motor skills. In 3EP, the anode was again secured over the center of the frontal pole of the subdominant hemisphere and the cathode was placed 2 cm above the ipsilateral mastoid process. The 6.25 cm^2^ electrodes were made of medical conductive rubber and placed in saline-soaked multilayer flannel cases. tDCS was administered with current intensities = 60–90 µA in the adolescents (current density = 0.001–0.014 mA/cm^2^) for 30–45 min in all subjects. The number of tDCS sessions performed varied from five to nine (stabilization of headache was the endpoint criterion), with each session separated by four to seven days. The primary outcome for treatment effectiveness was a ≥50% decrease in the number of days with headaches per month. Secondary outcomes included headache intensity (rated on a numerical rating scale (NRS) from 0–10) and duration, the dosages of analgesics taken, and depression/anxiety scale scores. Their results indicated a significant reduction in headache ratings (NRS pre: 5.11 ± 1.6 vs. NRS post: 2.11 ± 1.54), decreased number of days with a headache (10.32 ± 6.48 days vs. 4.11 ± 2.18 days), and decreased duration of headaches (4.57 ± 3.76 h vs. 2.45 ± 1.66 h). In addition, their data indicated that these effects were maintained for five to nine months on average. Furthermore, they also found that the efficacy of the stimulation depended on the location of the stimulating electrodes with 85% of the adolescent mTBI subjects reporting effective headache relief after two or three sessions using the 1EP montage. The authors postulated that this montage allowed them to influence both the frontal pole and, to a smaller degree, the mediobasal areas of the frontal lobes, from which a strong system of corticofugal fibers extends toward the reticular formation (RF) of the brainstem. Activation of mesencephalic RF is purported to be a primary tDCS treatment factor in people with posttraumatic headache related to mTBI. Indeed, a leading cause of such headaches might be a reduction of RF activation, leading to a disruption of the reticulo-cortico-subcortical neurodynamics [65,66] and the 1EP montage might have resulted in RF activation. Thus, by affecting these areas with tDCS, RF and thalamus activity might stabilize at an optimal level in these patients [67]. It was also noteworthy that the clinical efficacy of tDCS was comparable to typical pharmacological medications and other types of treatment, such as biofeedback and chiropractic manipulations [68,69,70]. Furthermore, the beneficial effects were more consistent and were present for longer durations than pharmacological therapies and presented with very few side effects. Accordingly, the authors concluded that tDCS was a promising treatment option for headaches from mTBI and various other etiologies.

Quinn, et al. [71] investigated whether anodal tDCS over the left dorsolateral prefrontal cortex (DLPFC) plus cognitive training altered cerebral blood flow (CBF) on pseudocontinuous arterial spin-labeling (pCASL) sequences in mTBI patients. Twenty-four subjects (15 male) with chronic mTBI with cognitive persistent post-traumatic symptoms were enrolled in the study. Subjects underwent 10 days of computerized executive function training combined with atDCS. All subjects had a diagnosis of mild or moderate TBI within the past 15 years and were randomly assigned to either active (*n* = 10 completed) or sham *n* = 14 completed) atDCS combined with executive function training tasks. The current intensity for the active group was 2 mA applied for 30 min (current density = 0.08 mA/cm2). All subjects trained their executive function for 30 min during stimulation. Each session consisted of 10 min of the AX Continuous Performance Task, which assesses response inhibition and proactive and reactive cognitive control [72], and 20 min of a modified multimodal (visual/auditory) N-back working memory task [73], counterbalanced over the 10 sessions. Cerebral perfusion imaging was accomplished via MRI scans performed during the baseline assessment, and on the day following completion of the stimulation + training protocol. Their results indicated similar cerebral perfusion and behavioral/cognitive test changes in both sham and active tDCS groups following the intervention. They noted that baseline CBF values were similar to other studies reporting decreased global CBF post-injury [74,75,76]. However, they noted a decrease in global CBF over time and that mood, attention, and executive function improved, potentially indicating enhanced cerebral efficiency. Changes in global CBF were weakly associated with one verbal learning test (*r* = −0.44), but this correlation did not survive Type I error correction (*p* = 0.03 uncorrected; *p* = 0.79, corrected). No other global CBF and objective cognitive performance or subjective mood associations were found. They postulated that the clinical condition and generalized perfusion represented a more complicated association than they had initially hypothesized. They also noted that adding tDCS to the cognitive training did not elicit any additional effects on global CBF. This finding was not novel as there is evidence that tDCS, which nominally influences primarily the brain targets under the electrodes, only induces perfusion changes in those specific target regions rather on the brain globally [77,78]. This concept was further supported in this study which also found that active tDCS was associated with increased CBF in the right inferior frontal gyrus, a brain area likely under the anode, whereas regional CBF of this same area was reduced in sham; although this finding again did not survive statistical correction. Nevertheless, this was interesting because of theoretical and empirical findings that have indicated different right prefrontal and right frontoparietal perfusions after mTBI [76,79,80,81]. The latter region is likely part of a network that has been associated with inhibition [82], visual attention, and emotion. Dysfunction of the former (i.e., right frontal areas) is also associated with anxiety [83], depression [84], impulsivity [85], somatization [86], and distractibility [87], all of which are potential comorbidities of chronic mTBI. The authors concluded that perfusion measured with pCASL might be a potential pathophysiologic target for symptom change assessment from cognitive training and/or tDCS in mTBI patients. However, it might be necessary to obtain both CBF and metabolic activity (i.e., positron emission tomography) measures to better understand how the brain adapts to injury and responds to training [88].

The symptoms of mTBI include non-motor symptoms such as headache, loss of consciousness, and memory loss, but there are also motor symptoms that include balance impairment, lack of motor coordination, and decreased dynamic motor function [5,89], especially in acute injury. These symptoms often make it difficult for people with mTBI to returning to sport and leisure activities or to perform at pre-mTBI levels. Although studies have reported that tDCS might have therapeutic effects on motor function in patients with neurological disorders such as multiple sclerosis (reviews in [90,91]), no study has assessed the effect of atDCS on balance in humans with repetitive mTBI. Still, animal models might serve s a foundation for such investigations. Importantly, human brains and rat brains are anatomically similar and studying human brain diseases via rodent investigations may be informative [92]. Leveraging the close evolutionary and genomic similarities to humans, the intricacy and conviviality of the animal, and the ease of physiological and behavioral measurements, the rat represents a key preliminary model for non- and invasive brain stimulation research [93]. Accordingly, Park, et al. [93] investigated if atDCS over the primary motor cortex (M1) will improve balance and gait function in a repetitive mTBI rat model. Sixty-five rats were randomly assigned to either a tDCS group or a control group. To simulate repetitive mTBI in rats the authors induced mTBI for three consecutive days via a weight-drop device (rodents anesthetized during induction). mTBI was confirmed via lack of structural/pathological changes via MRI and histochemical analysis. tDCS was administered for 30 min via 10 mm diameter (0.785 cm^2^ contact area) cup electrode (anode) secured over the left M1 with a 0.2 mA intensity (current density = 0.255 mA/cm^2^). The tDCS group received a single session of atDCS over the left M1 24 h after the third weight drop day (Day 4; animal anesthetized during tDCS administration) and the control group received no treatment but was still anesthetized on Day 4. The outcomes were changes in TMS-induced motor-evoked potential (MEP), a foot-fault test (balance control), and a rotarod test (postural orientation) evaluated before mTBI (Day 1), after the last weight drop (Day 3), and after tDCS (Day 4), with similar time-based evaluations for the control group. The findings indicated that atDCS administered the day after repetitive mTBI induction increased the amplitude of MEP, decreased (trend only) the foot-fault ratio, and significantly improved rotarod duration. The authors discussed that the stimulated area was composed of the primary motor and premotor cortices and postulated that atDCS over these areas generated an electric field that polarized the underlying neuronal populations and modulated the resting membrane potential in their mTBI rats. Subsequently, the corticospinal tract might have been activated and induced the balance and gait improvements. Moreover, their results suggested that mTBI reduced the number of motor units recruited in corticospinal excitability and that this acute deficiency might be restored by atDCS over these areas. This seems plausible because the replacement of lost fibers might be facilitated by the concurrent excitement of motor units [94]. These findings [93] provide preliminary evidence for tDCS as a promising tool to modulate brain network function and, subsequently, supraspinal motor control and might provide a translational platform to bridge human and animal studies and establish new therapies for repetitive mTBI. Still, some methodologies unique to such animal models warrant consideration. Specifically, because the rats in the Park, et al. [93] study were anesthetized during both the weight drops and tDCS, the potential impact of anesthesia on the study outcomes or efficacy of stimulation cannot be overlooked. Additionally, their rats experienced targeted, repetitive (i.e., daily for three sessions) mTBI and stimulation was administered over the same targeted/damaged brain area. Both mTBI and tDCS in humans are inherently less precise and their brain injuries may not occur on a predictable, or even on a repeated, schedule. Thus, researchers using both humans and animals should carefully consider if the proposed study group has experienced single or repeated mTBI and how the short- and long-term effects might differ by brain injury frequency.

## 4. Discussion and Perspectives

We performed a comprehensive review of the effect of tDCS on cognitive and motor impairment within the mTBI population. Based on the review, there is limited evidence of tDCS improving cognitive and motor performance. Surprisingly, there were only three studies that used tDCS in mTBI, which highlights an urgent need for more research to provide additional insights into ideal therapeutic brain targets and optimized stimulation parameters.

The frontal poles [64], DLFPC [71], and M1 [93] were the tDCS brain targets in the reviewed studies and the choice of the tDCS target brain target should coincide with the study outcomes and be relevant to the studied population. For example, the DLPFC is the important role that this site exerts in cognitive function and some studies have shown that DLPFC is associated with attention and working memory function [45,95]. Indeed, this region is a hub of the executive functions required to coordinate and integrate different cognitive processes [96]. Therefore, researchers might logically choose this brain target when investigating cognitive outcomes. For motor outcomes, other relevant brain areas might be stimulated. For example, Park, et al. [93] applied tDCS over M1 to improve balance and posture in their rat model of mTBI. However, balance and posture are influenced by various interacting networks, including the spinal cord, cerebellum, cortex, and brainstem [97,98]. Of these, some might argue that the cerebellum would be an ideal tDCS target for balance and postural control. For example, Yosephi, et al. [99] suggested that bilateral stimulation of the cerebellar hemispheres was more effective than stimulating M1 to improve balance in older adults with a high fall risk. However, a previous study by Sussman, et al. [100] found that mTBI was associated with white and gray matter volume reduction and cortical thinning in areas that included M1, but such brain injury left the cerebellum unaffected. Furthermore, a recent study highlighted the critical interactions between M1 and the cerebellum for effective motor function [101] and there is evidence that the effects of tDCS can influence brain areas in remote locations [102,103,104]. Thus, tDCS researchers should carefully consider the potential direct and indirect influences of the stimulation and select brain targets that will most relevantly affect the studied population.

However, the ideal tDCS brain target may differ for each individual, especially in mTBI. To make the clinical application of tDCS for cognitive impairment more robust, it is necessary to consider the heterogeneity of brain injury sites within this population. Thus, the results obtained from tDCS may vary substantially and would be reflected in brain activity changes that might be assessed via neuroimaging and neuropsychological tests. Furthermore, in the two extracted human studies [64,71], the target patients were identified based on symptoms of cognitive impairment post-TBI as opposed to being categorized by objective measures, such as conventional brain imaging or known brain lesion, which may not be possible in this population [19,20]. Understandably, from the standpoint of rehabilitating the entire population, it may not be appropriate not to select patients based on their known lesions. However, for tDCS to be established as a rehabilitation method for cognitive and motor impairment, it is necessary to systematically select patients from their brain function imaging (e.g., diffusion tensor imaging, PET imaging). In addition, more evaluations using functional brain imaging to predict prognosis and identify tDCS responders would be beneficial. For example, molecular imaging techniques that examine functional processes within the brain, such as [^18^F]fluorodeoxyglucose and positron emission tomography (FDG-PET), can detect changes after mTBI and over time. Such techniques are an increasingly viable option as recent technological improvements in the resolution of PET systems, the integration of PET with structural magnetic resonance imaging (MRI), the availability of normal healthy human databases, and commercial image analysis software are contributing to the growing use of molecular imaging in basic science research and advancing this modality in clinical settings [88,105].

The intra- and inter-individual variabilities in “ideal” parameters for electrical current application, stimulation targets, and responses are some of the major concerns prohibiting the widespread use of tDCS in real clinical settings. As pointed out in a review by Rudroff, et al. [105], it is a challenge to determine optimal treatment procedures to adapt the configurations to the complex shapes and dramatic conductivity differences among various tissues (e.g., scalp, skull, cerebrospinal fluid, gray matter, etc.). In other words, placing the tDCS electrodes directly over the targeted brain area does not guarantee that those areas will be modulated (excitation or inhibition) as expected [106]. Computer simulations and tDCS modeling studies have helped identify the theoretical behavior of induced electrical currents [107], however, they have many limitations. For example, they necessarily make assumptions about the conductivity of the underlying tissues (and the relevancy of such tissues), but different conductive values can lead to highly variable results in electrical field magnitudes [108,109]. Other factors that might alter the electrical field include registration procedure errors, anatomic variations [110], functional connectivity, and inter-individual variability (e.g., age, gender, hormones, neurotransmitter levels, neuroanatomy [111]). Consequently, it is essential to routinely combine tDCS with human neuroimaging via structural/functional magnetic resonance imaging (sMRI/fMRI) or PET to investigate cerebral blood flow and metabolism mechanisms. Specifically, FDG-PET imaging can provide a comprehensive (e.g., whole brain) image, it is ideally suited to not only investigate the effects of tDCS in areas directly under the stimulation electrodes but also in remote or functionally connected brain areas [105].

One factor, that contributes significantly to the high inter-subject variability of tDCS is biological sex differences [111,112,113]. For example, it has been shown that female hormones endogenously influence cortical excitability [114], with progesterone levels driving increases in cortical inhibition and estrogen enhancing cortical excitability. Accordingly, tDCS applied in phases of the menstrual cycle when estradiol is high may result in cortical overexcitation and unpredictable or unwanted outcomes [115]. The enlightenment of ideal tDCS methodologies specific to each sex (e.g., low intensities for women and high intensities for men, or vice versa) would represent a vital foundation for individualized tDCS applications. However, sex-related differences represent only one of a myriad of issues (e.g., age, handedness, cognitive ability, neurological and psychiatric disorders, medications, recreational drugs, prior exposure to brain stimulation, electrode configurations, stimulation parameters, task dependency) suspected to contribute to the high variability in tDCS outcomes [111]. Furthermore, optimal protocols for interventions coupling tDCS and physical therapy are yet to be defined. Factors such as the tDCS technique used and its parameters (e.g., polarity, intensity, duration), the target area, the type of physical training performed, and its timing in relation to stimulation (before, during, or after) can all influence the therapeutic outcomes. Furthermore, these factors will likely need to be individually tailored based on patient-specific considerations such as the time since brain injury and the specific anatomical and neurophysiological derangements unique to each patient. Therefore, the need for further research in mTBI cannot be overemphasized. It is currently questionable whether standardized tDCS applications, to treat mTBI and in general, are feasible, at least in the near future. Furthermore, it is practically impossible to include all characteristics of every individual into clinical trial study designs to obtain homogenous samples and tDCS tailored to individual participants is a more likely solution to address response heterogeneity.

## Data Availability

No new data were created or analyzed in this study. Data sharing is not applicable to this article.
